# RNA sequencing and *de novo* assembly of *Solanum trilobatum* leaf transcriptome to identify putative transcripts for major metabolic pathways

**DOI:** 10.1038/s41598-018-33693-4

**Published:** 2018-10-18

**Authors:** Adil Lateef, Sudheesh K. Prabhudas, Purushothaman Natarajan

**Affiliations:** Department of Genetic Engineering, School of Bioengineering, SRM Institute of Science and Technology, Kattankulathur, 603203 India

## Abstract

*Solanum trilobatum* L. is an important medicinal plant in traditional Indian system of medicine belonging to Solanaceae family. However, non-availability of genomic resources hinders its research at the molecular level. We have analyzed the *S. trilobatum* leaf transcriptome using high throughput RNA sequencing. The *de novo* assembly of 136,220,612 reads produced 128,934 non-redundant unigenes with N50 value of 1347 bp. Annotation of unigenes was performed against databases such as NCBI nr database, Gene Ontology, KEGG, Uniprot, Pfam, and plnTFDB. A total of 60,097 unigenes were annotated including 48 Transcription Factor families and 14,490 unigenes were assigned to 138 pathways using KEGG database. The pathway analysis revealed the transcripts involved in the biosynthesis of important secondary metabolites contributing for its medicinal value such as Flavonoids. Further, the transcripts were quantified using RSEM to identify the highly regulated genes for secondary metabolism. Reverse-Transcription PCR was performed to validate the *de novo* assembled unigenes. The expression profile of selected unigenes from flavonoid biosynthesis pathway was analyzed using qRT-PCR. We have also identified 13,262 Simple Sequence Repeats, which could help in molecular breeding. This is the first report of comprehensive transcriptome analysis in *S. trilobatum* and this will be an invaluable resource to understand the molecular basis related to the medicinal attributes of *S. trilobatum* in further studies.

## Introduction

*Solanum trilobatum* L. is one of the important medicinal plants belonging to family Solanaceae, more commonly available in Southern part of India. *S. trilobatum* is a prickly diffuse, bright green perennial herb, 2–3 m in height, mostly found in dry places, and grows like a weed along roadsides and wastelands^1^. In traditional Indian system of medicine, it is broadly used to treat many respiratory disease conditions. Its extract is used to treat conditions like chronic bronchitis and tuberculosis^2,3^. A pilot study, on clinical efficacy of *S. trilobatum* and *Solanum xanthocarpum*, confirmed the usefulness of these herbs in bronchial asthma. The reports confirmed its response to be equivalent to deriphylline but less than that of salbutamol, which is the preferred choice of drugs for the treatment of bronchial asthma^4,5^. *S. trilobatum* is also reported to have shown different activities like anti-oxidative activity, hepatoprotective activity^6–8^, anti-inflammatory activity^9^, anti-microbial activity^10^ and anti-tumoral activity^11^. The ethanol extract of *S. trilobatum* have shown a hypoglycaemic effect in alloxan-induced diabetic rats through antioxidant defense mechanism^12^. The majority of secondary metabolites, attributed to medicinal properties are mainly present in its leaves, including alkaloids such as soladunalinidine and tomatidine. *S. trilobatum* is reported to contain various chemical compounds as sobatum, β-solamarine, solasodine, solaine, and diosogenin^13,14^. Moreover, sobatum, purified from *S. trilobatum*, is found to be effective in suppressing drug-induced toxicity in rats^15^. Despite the well-established role of *S. trilobatum* in traditional Indian medicine, the genetics and genomics of this medicinal plant are least explored. As for the genomic resources, only 56 nucleotide sequences are available at National Center for Biotechnology Information (NCBI) database for *S. trilobatum* (as accessed on February 1, 2018). An in-depth study of *S. trilobatum* transcriptome is needed for analysis and characterization of its functional genes. This would help to achieve large-scale production of drugs via molecular breeding, transgenic technology, and metabolic engineering. RNA Sequencing-based *de novo* assembly is a well-developed approach to understanding transcriptomes of non-model plants with limited genomic information. Moreover, RNA-Seq is a cost-effective tool, offers much data with better coverage and sufficient sequence depth for *de novo* assembly of transcriptomes. In the past few years, there has been a significant increase in utilizing RNA-Seq for discovery and identification of functional genes involved in the biosynthesis of active compounds in plants^16^. In this study, we report the transcriptome from the leaf of *Solanum trilobatum* using high throughput next-generation sequencing for the first time. The high-quality reads were *de novo* assembled into unique transcripts, which were then extensively evaluated and annotated to identify the putative pathways and genes responsible for its medicinal properties.

## Materials and Methods

### Plant material and RNA isolation

*Solanum trilobatum* L. collected from Guduvanchery, Kancheepuram district, Tamil Nadu was taxonomically identified by the Centre for Floristic Research, Madras Christian College, Tambaram, Chennai, with field no. 523. The mature leaves from top and middle parts of the healthy plant during its flowering stage were collected and used for total RNA extraction immediately after collection using modified CTAB method^17^. The extracted total RNA was treated with DNase A and purified using RNeasy MinElute clean up kit (Qiagen Inc., GmbH, Germany). The quality was assessed using NanodropLite spectrophotometer (Thermo Scientific, Wilmington, Delaware, USA) and Qubit 2.0 (Invitrogen, Carlsbad, California, USA). The RNA integrity value was measured using Bioanalyzer 2100 (Agilent Technologies, Santa Clara, California, USA). The purified total RNA was used for sequencing library preparation.

### Library preparation and illumina sequencing

The total RNA was made ribosomal RNA free using Ribo-Zero rRNA removal kit (Illumina Inc., Singapore) and the remaining fraction was purified and eluted. The purified RNA was disrupted into short fragments using fragmentation buffer; these short fragments are used as a template for first strand cDNA synthesis using superscript II reverse transcriptase (Invitrogen, Carlsbad, California, USA) followed by second strand synthesis and purification. The purified double-stranded cDNA was polyadenylated, and adapter-ligated for paired-end library preparation. The adaptor primers were used for amplification of the library for the enrichment of the cDNA fragments. Caliper LabChip GX using HT DNA High Sensitivity Assay Kit (Caliper Life Sciences Inc., USA) was used for library quality assessment. The library was hybridized on a flowcell, and clonal clusters were generated on cBOT using TruSeq PE Cluster Kit v3-cBot-HS (Illumina Inc., USA). Sequencing was carried out on Illumina Hiseq. 2500 using TruSeq v3-HS kit to generate 100 bp paired-end reads (Illumina Inc., USA).

### *De novo* assembly and clustering

The raw paired-end reads were quality assessed by FastQC v0.11.2^18^. Pre-processing of raw reads was performed with Cutadapt v1.7.1^19^ and Sickle v1.33 tools^20^ for adapter trimming and quality filtering respectively. Reads with Phred score >=30 were retained. The filtered reads were further used for transcriptome assembly using Trinity^21^. Trinity, a *de novo* assembler consists of Inchworm, Chrysalis, and Butterfly modules, which are applied sequentially to process RNA-seq raw reads into full-length transcripts. The process begins with Inchworm which generates full-length transcripts from raw reads based on default k-mer values. The Chrysalis clusters the contigs generated by Inchworm and prepares de bruijn graph for each cluster. Finally, butterfly processes individual graphs reporting full-length transcripts for alternatively spliced isoforms. The redundancy of the assembled contigs was removed using CD-HIT v4.5.4^22^.

### Assessment of gene completeness

The gene completeness analysis was performed by using the TRAPID tool (http://bioinformatics.psb.ugent.be/webtools/trapid). The analysis was carried out by comparing the unigene transcripts against PLAZA 2.5 green plants clade database^23^ with an E-value of <1E-5 for significant similarity search and annotation of unigenes. The completeness of unigenes was assessed by considering one or more hits in TRAPID database^24^ for “full length”, “quasi full length” or “partial” based on length of the open reading frame (ORF).

### Functional annotation and classification

The *de novo* assembled sequences of *S. trilobatum* were compared against plant non-redundant (nr) protein database at National Centre for Biotechnology Information (NCBI) using BLASTX tool from stand alone BLAST+ package with an E-value parameter not greater than 1E-05 for identification of best significant match. The BLASTX results were further imported to Blast2GO suite^25^ for retrieving Gene Ontology (GO) terms of assembled unigenes and for mapping, the annotation was further continued with unique enzyme codes (EC) and Kyoto Encyclopedia of Genes and Genomes (KEGG) maps. Moreover KEGG Automated Annotation Server (KAAS) was also used for pathway mapping in addition to Blast2GO. GO terms are precisely defined as controlled vocabulary which can be used to describe functions of genes or gene products. The assembled transcripts based on the retrieved GO terms were classified into three categories viz. Biological process, Molecular function, and Cellular component. The pathway maps were determined from KEGG database with an E-value of 1E-05.

### Prediction of transcription factor families

The prediction of transcription factor (TF) families, in *S. trilobatum* transcriptome, was done using Plant Transcription Factor Database^26^ (plnTFDB v4.0; http://planttfdb.cbi.pku.edu.cn/prediction.php).

### Identification of simple sequence repeats (SSRs)

The MicroSAtellite Identification tool (MISA) was used for identification of SSRs from assembled unigenes of *S. trilobatum*^27^. The parameters were set to identify perfect di-, tri-, tetra-, penta- and hexa nucleotide motifs with a minimum thresholds of 6, 5, 5, 5 and 5 repeats respectively.

### Transcript quantification

The estimation of unigene abundance was determined using RNA-Seq by Expectation-Maximization (RSEM) tool, which quantifies transcript level abundance from RNA Seq data. RSEM first generates and preprocesses a set of reference transcript sequences and then aligns reads to reference transcripts followed by estimation of transcript abundances. RSEM calculates fragments per kilo base per million (FPKM) and transcripts per million (TPM) values for the assembled individual unigenes from *S. trilobatum*^28^. FPKM and TPM values are calculated to understand the expression levels of unigenes involved in biosynthetic pathways of secondary metabolites.

### Validation by reverse transcription PCR

In order, to experimentally validate *de novo* transcriptome assembly, some of the assembled unigenes of *S. trilobatum* which share sequence similarity to various secondary metabolite biosynthetic pathway genes were selected for reverse transcription-PCR. All the primers were designed from final assembled sequences, and actin (house keeping gene) was used a positive control. The primer sequences are given in Supplementary Table S1. The reverse transcription-PCR products were electrophoresed on 1% agarose gel.

### Gene expression analysis by qRT-PCR

The quantitative gene expression analysis was performed using QuantStudio 5 Real-Time PCR System (Thermo Scientific, Wilmington, Delaware, USA) and QuantiNova SYBR Green PCR Kit (Qiagen Inc., GmbH, Germany). For each primer pair, a control reaction without a template was also included. Elongation factor 1-alpha (*ef1a*) from *Solanum trilobatum* was used as an internal reference gene for normalization and estimation of gene expression. The data from qRT-PCR data was analysed using comparative Ct (2^−ΔΔCt^) method. Fold change in gene expression was calculated as 2^−ΔΔCt^ using ΔCt values^29^. All the experiments were repeated using three technical and two biological replicates. Gene specific oligonucleotides used for qRT-PCR analysis are provided in Supplementary Table S2.

## Results and Discussion

### Illumina paired-end sequencing and *De novo* assembly

**Figure 1:**
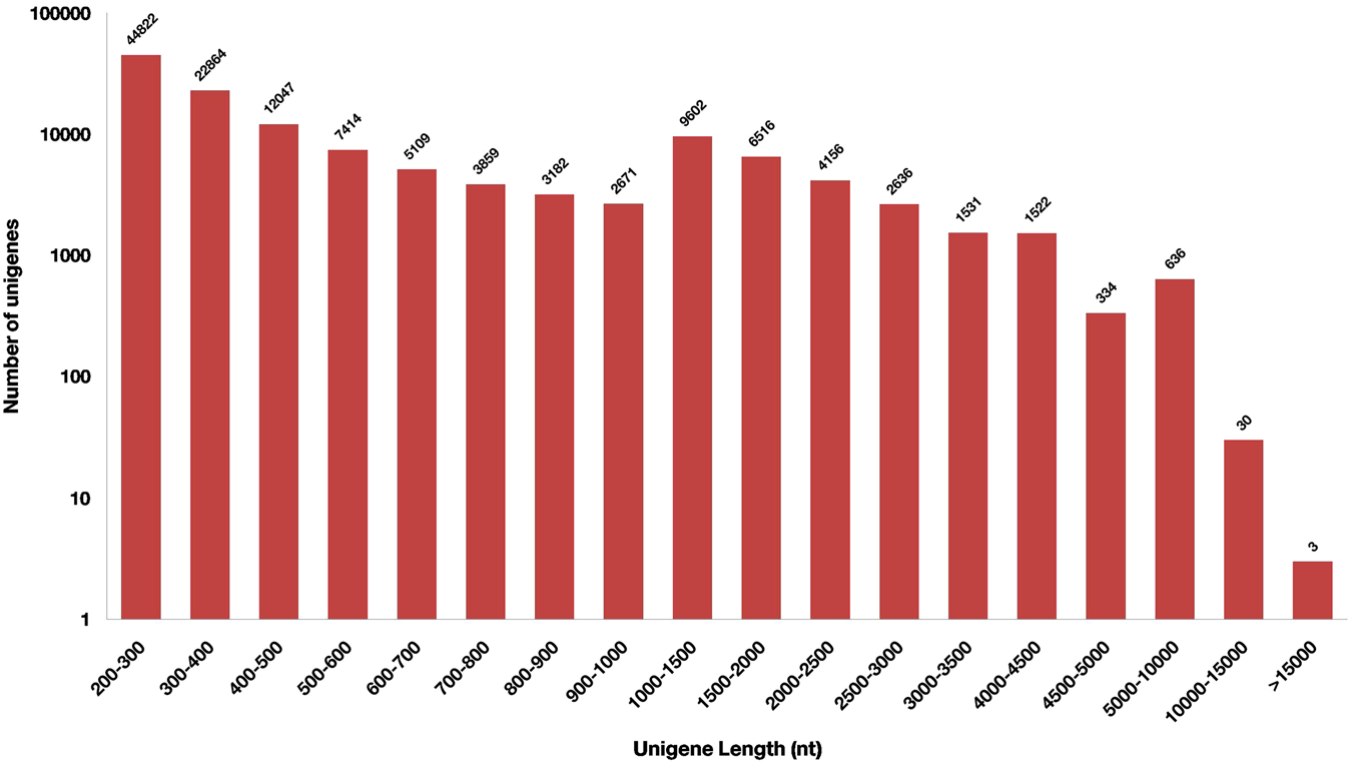
Overview of unigene length distribution from *Solanum trilobatum* leaf transcriptome.

A total of 136,220,612 raw reads were generated from *Solanum trilobatum* leaf transcriptome that accounts for about 35.4 GB of paired-end sequencing data. The raw data were deposited at National Centre for Biotechnology Information (NCBI) Short Read Archive (SRA) database under the accession number SRP132765. The pre-processing of raw reads was done for removal of adaptor sequences and low-quality reads (Phred score <30) and a total of 124,413,306 clean reads (Phred score >=30) with GC content 39.21% were retained. Trinity assembler was employed for *de novo* assembly of short read sequences. A total of 144,580 assembled transcripts were generated with a mean size of 823 bp. The assembled transcripts were further clustered, using CD-HIT, into 128,934 unigenes with a mean length of 745 bp and N50 contig length of 1347 bp. The transcriptome assembly details are given in Table 1. Among unigenes, the minimum length was 201 bp and maximum length 19956 bp. The length distribution of the unigenes is given in Fig. 1.

**Table 1:** Summary of Illumina paired-end sequencing and *de novo* assembly of *Solanum trilobatum*.

Particulars	Numbers
Number of raw reads	136220612
Number of clean reads	124413306
No. of bases (after processing)	11510033710
Mean Phred Score	37.45
Total transcripts/unigenes	128934
Percentage of successful assembly from raw reads	89.91%
Total length (bases)	96142297
Average length (bases)	745.67
Median contig length	382
Max length (bases)	19956
Min length (bases)	201
GC (%)	39.21
Contig N50 (bases)	1347

### Assessment of gene completeness

Gene completeness analysis using TRAPID tool revealed the presence of 17521 (13.6%) full-length unigenes, 9517 (7.4%) quasi full length and 12433 (9.6%) partial coding unigenes. Moreover, there were 89463 (69.4%) unigenes that did not match to any protein in PLAZA green plants clade database.

### Functional annotation of unigenes

**Figure 2:**
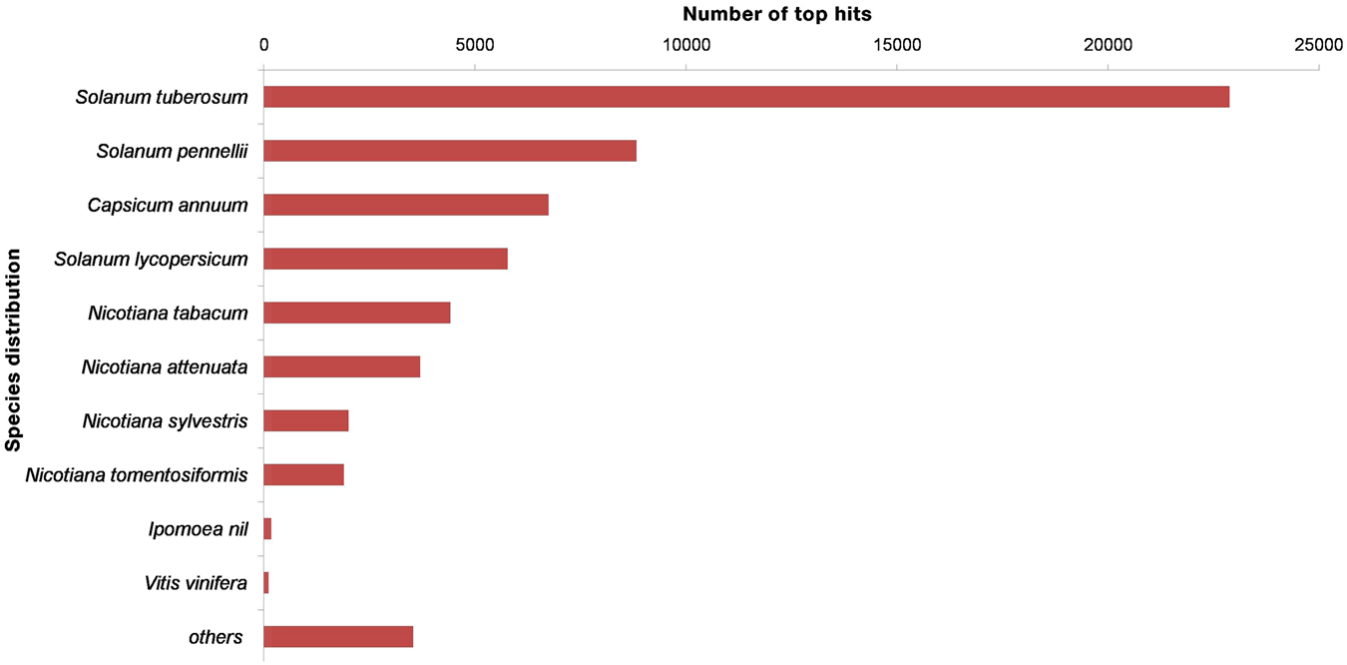
BLASTX top hit species distribution of *Solanum trilobatum* unigenes against plant nr database.

The assembled unigenes of *S. trilobatum* were annotated for sequence similarity search and comparison using BLASTX against plant non-redundant protein database at NCBI with an E-value cut off 1E-5. The BLASTX results showed 60,097 annotated unigenes, and 68,837 remained non-annotated unigenes out of 128,934 assembled transcripts. Among annotated unigenes, 1.5% were uninformative hits (e.g., Predicted: Uncharacterized or hypothetical proteins) due to inadequate *S. trilobatum* genome information in public databases. BLASTX results were imported to BLAST2GO suite for further annotation. The unigenes showed top-hit species similarity with *Solanum tuberosum (38.08%), Solanum pennellii (14.69%), Capsicum annuum (11.22%), Solanum lycopersicum (9.61%), Nicotiana tabacum (7.36%)* and others (Fig. 2). The results indicate that *S. trilobatum* is more closely related to *S. tuberosum*. The annotations against proteins in Pfam database showed 35,141 significant hits. Moreover, 61986, 30427, and 14490 unigenes were annotated against the Uniprot, GO and KEGG databases respectively (Table 2). BLAST2GO is a preferable tool for large-scale functional annotation and data mining for sequencing data of non-model species. BLASTX results were imported to BLAST2GO tool for mapping to retrieve GO terms and then to retrieve EC numbers. The KEGG pathway annotation for assembled unigenes of *S. trilobatum* was performed using BLAST2GO and KAAS against KEGG database to unravel molecular interaction networks and metabolic pathways. (Supplementary Table S3).

**Table 2:** Unigene homology searches against biological databases.

Database	Unigene	Percentage
Nr	60097	46.61%
Pfam	35141	27.25%
KEGG	14490	11.23%
GO	30427	23.60%
Uniprot	61986	48.07%

### Functional classification of unigenes

**Figure 3:**
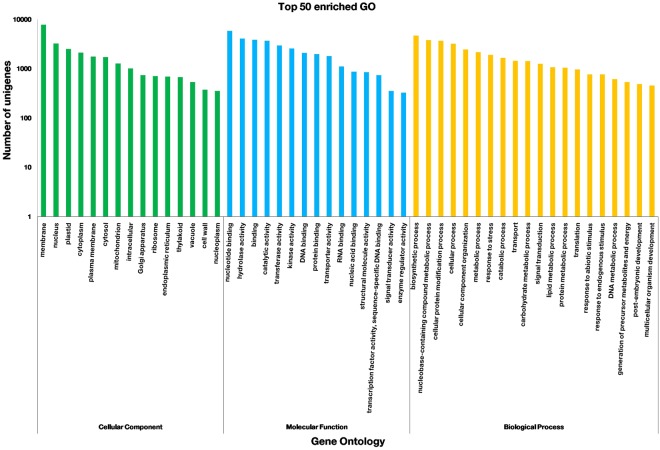
GO classification of *Solanum trilobatum* unigenes. The Histogram shows the results of unigene classification under three major categories of GO terms.

Gene Ontology (GO) classification was used to classify assembled unigenes based on annotations. Using BLAST2GO based on GO annotation, 30427 unigenes were assigned to one or more GO terms, which were allocated to three major categories and 97 subcategories. In biological function, category 38,714 unigenes were assigned to 44 classes. This category includes proteins highly involved in the biosynthetic process (4671 unigenes), nucleobase-containing compound metabolic process (3808 unigenes), cellular protein modification process (3704 unigenes) and cellular process (3201 unigenes). In molecular function, category 34,917 unigenes were assigned to 26 classes and includes proteins highly encoded in nucleotide binding (5826 unigenes), hydrolase activity (4093 unigenes), binding (3847 unigenes) and catalytic activity (3698 unigenes). Cellular components were grouped into 27 classes with most assignments to a membrane (7773 unigenes), nucleus (3242 unigenes), plastid (2519 unigenes) and cytoplasm (2114 unigenes) (Fig. 3) (Supplementary Table S4).

### Biological pathway analysis

**Figure 4:**
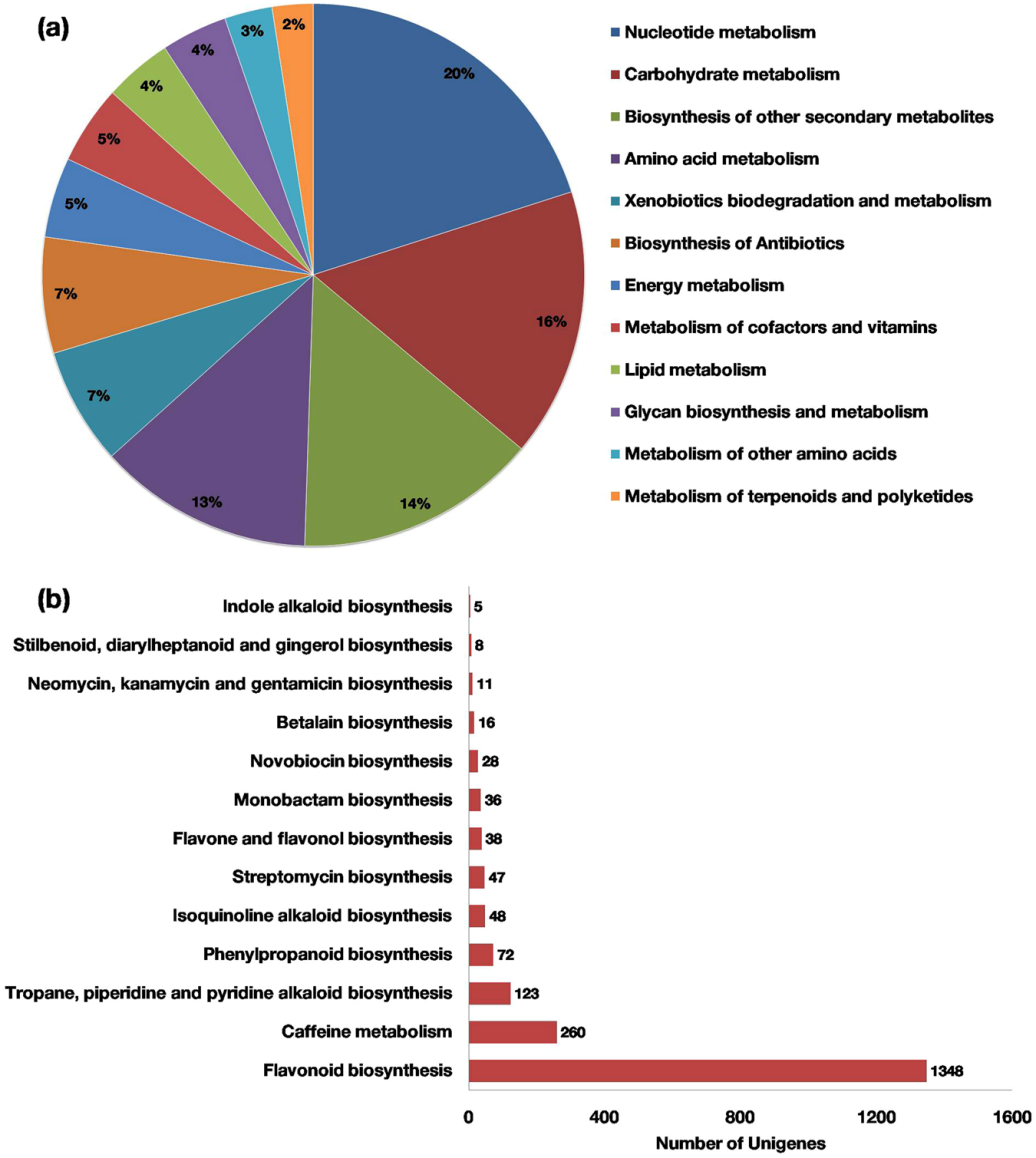
KEGG pathway unigene assignments. (**a**) Based on metabolic pathway. (**b**) Based on secondary metabolite biosynthesis.

The active biochemical pathways in *S. trilobatum* were identified by mapping the unigenes against KEGG pathway database using KAAS and BLAST2GO. In total, 14,490 (11.23%) unigenes were annotated using KEGG database and assigned to 138 pathway maps. There were 14,212 unigenes assigned to metabolic pathway category including nucleotide metabolism, carbohydrate metabolism and biosynthesis of secondary metabolites with 2849 (20%), 2277 (16%) and 2050 (14%) unigene respectively (Fig. 4a). The unigenes representing secondary metabolism were further divided into 17 sub-categories, where in “flavonoid biosynthesis” pathway was found to be encoded by highest number of unigenes (1348 unigenes) followed by “caffeine metabolism” (260 unigenes) “tropane, piperidine and pyridine alkaloid” biosynthesis (123 unigenes), “phenylpropanoid biosynthesis”(72 unigenes), “isoquinoline alkaloid” biosynthesis (48 unigenes) (Fig. 4b).

### Secondary metabolite biosynthesis

#### Phenylpropanoid biosynthesis genes

**Figure 5:**
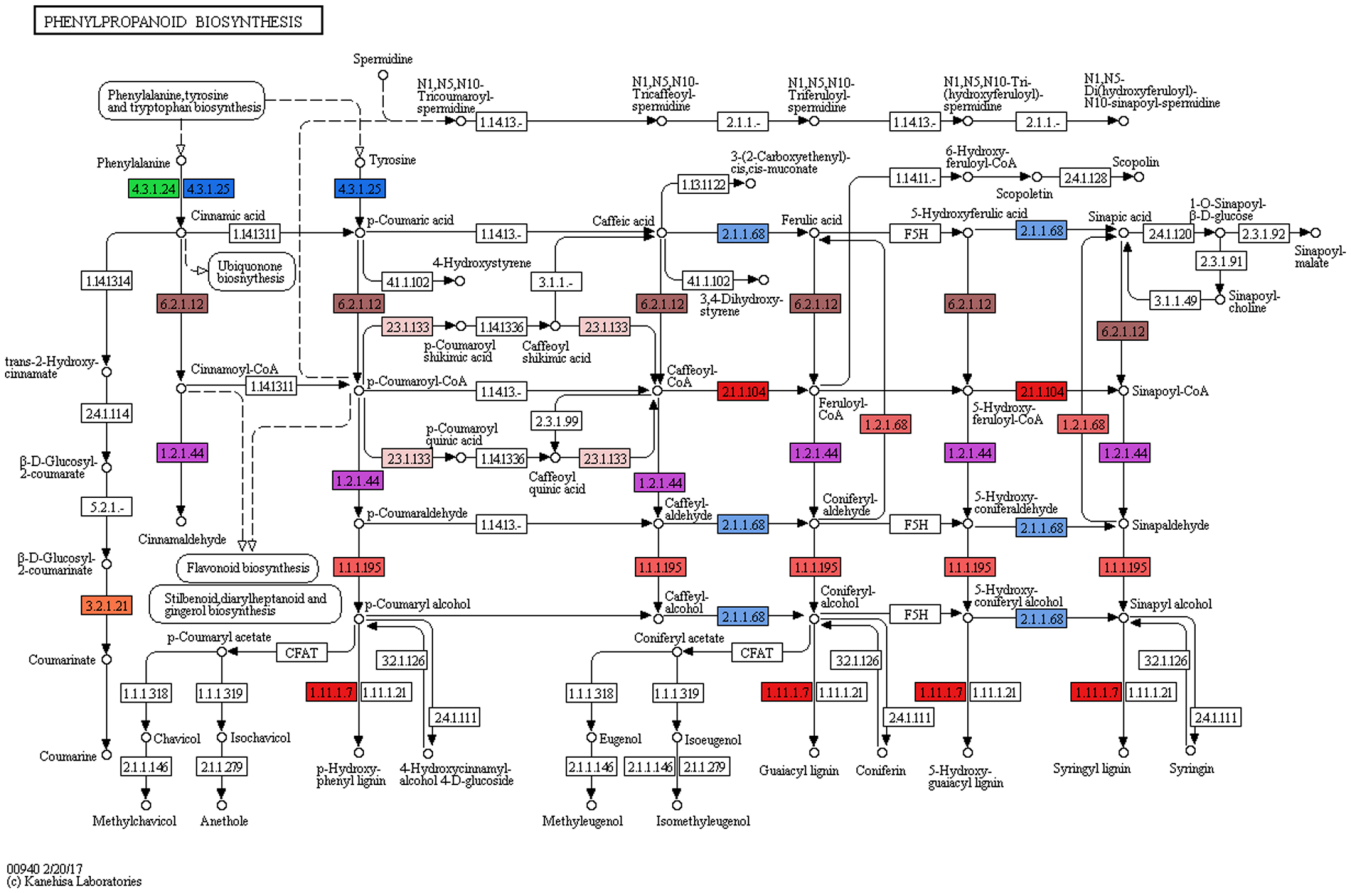
Phenylpropanoid biosynthesis pathway from *Solanum trilobatum* by KEGG analysis showing the different identified enzymes (one color for each Enzyme Code or EC). KEGG pathway map 00940 is adapted here from http://www.kegg.jp/kegg/kegg1.html. The KEGG database has been described previously^60–62^.

Phenylpropanoids comprise a large group of plant-based natural compounds derived from phenylalanine^30^. Phenylpropanoids are responsible for plant responses to biotic and abiotic stimuli^31^. Phenylpropanoid biosynthesis starts with the formation of Cinnamic acid from Phenylalanine, which gets converted into Cinnamoyl-CoA, p-Coumaryl-CoA, p-Coumarylquinic acid, Caffeoyl quinic acid, Caffeoyl-CoA, Feruloyl-CoA, and Sinapoyl-CoA. Caffeoyl quinic acid (Chlorgenic acid) is a well established soluble phenylpropanoid in Solanaceae and also plays a role as defense compound or as a potential antioxidant^32^. In this study, KEGG analysis revealed the presence of 11 genes which are involved in the biosynthesis of various compounds of this pathway. The major genes identified were Phenylalanine ammonia lyase (EC: 4.3.1.24, EC: 4.3.1.25), Trans cinnamate-4-monooxygenase (EC: 1.14.13.11), Cinnamoyl-CoA CoA reductase (EC: 1.2.1.44) and 4-Coumarate CoA ligase (EC: 6.2.1.12). The presence of all these enzymes compliments the phytotherapeutic properties of *S. trilobatum* (Fig. 5).

#### Analysis of flavonoid biosynthesis genes

The coloring pigment of most flowers, fruits, and seeds are flavonoids. They are widely distributed in plants and classified into 6 major subgroups: chalcones, flavones, flavonols, flavandiols, anthocyanins and proanthocyandins, one more subgroup is found in some species, the aurones. Flavonoids are synthesized through Phenylpropanoid pathway, by transformation of phenylalanine to p-Coumaryl-CoA, which actually enters into the flavonoid biosynthesis pathway. The first enzyme specific to this pathway is Chalcone Synthase, which produces chalcone scaffolds used for the formation of other flavonoids^33^. In the present study, Chalcone synthase (EC: 2.3.1.74) and Chalcone isomerase (EC: 5.5.1.6) enzymes were identified and are responsible for the formation of Naringenin from p-Coumaroyl-CoA. The enzymes required for conversion of Naringenin to produce apiforol by flavonone-4-reductase (EC: 1.1.1.234), eriodictyol by flavonoid-3′5′-hydroxylase (EC: 1.14.1388) and dihydrotricetin by flavonoid 3′-monooxygenase (EC: 1.14.13.88) were also identified. Moreover, the enzyme 6′-deoxychalcone synthase (EC: 2.3.1.170) responsible for converting p-Coumaroyl CoA to Isoliquiritigenin for production of butein is also identified in our dataset where Chalcone isomerase further converts butein to butin. The flavonone 4- reductase also acts on eriodictyol to produce a bioactive compound luteoforol. The enzyme Naringenin-3-dioxygenase (EC: 1.14.11.9), found in our data set, acts on pinocembrin to produce pinobanksin which is further acted by Flavanol synthase (EC: 1.14.11.23) to convert it into galingin, Pinocembrin also gets converted to Pinostrobin. Naringenin-3-dioxygenase acts on liquiritigenin to produce garbanzol and also acts on eriodictoyl to produce dihydroquercetin or taxifolin which is further converted to quercetin by Flavonol synthase enzyme were identified in *S. trilobatum* leaf transcriptome dataset. Moreover, Naringenin 3-dioxygenase acts on dihydrotricetin to convert it into dihydromyricetin which is finally converted to myrecitin by Flavonol synthase. A similar set of flavonoid pathway genes has been already reported in leaf transcriptomes of endangered medicinal plant *Chlorophytum borivilianum*^34^ and also in the leaf tissue of medicinal plant *Phyllanthus amarus*^35^. These reports support our findings in the present dataset and also suggest how flavonoid pathway is complimenting for medicinal properties of *S. trilobatum*. The current study identified the genes in the biosynthesis of various flavonoids like butein, butin, pinostrobin, naringenin, galingin, garbanzol, dihydrofisetin (futin), eriodictyol, homoeriodictyol, kaempferol, apiforol, luteoforol, myricetin, dihydroquercetin (taxifolin) and cyanidin.

**Figure 6:**
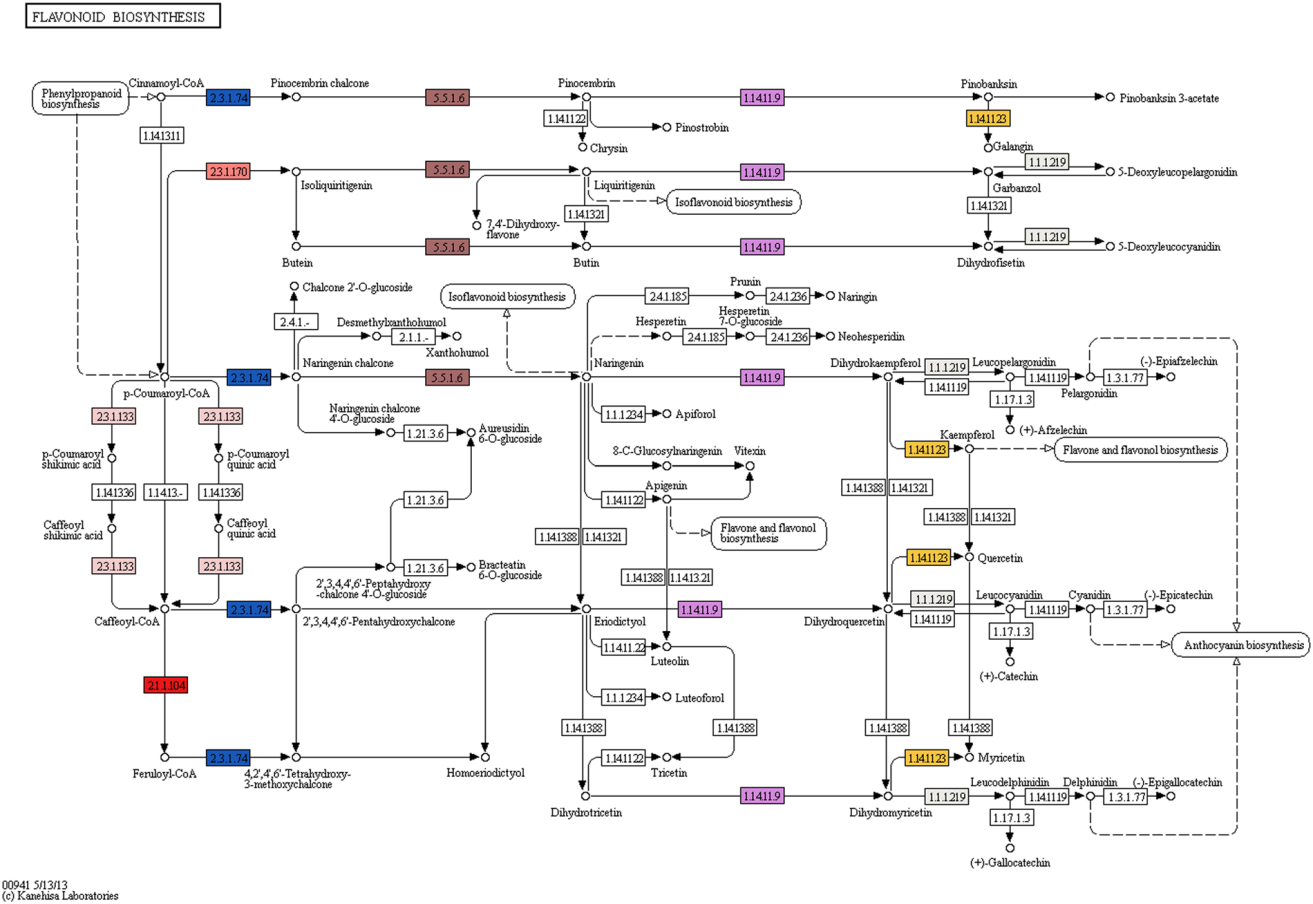
Flavonoid biosynthesis pathway from *Solanum trilobatum* by KEGG analysis showing the different identified enzymes (one color for each Enzyme Code or EC). KEGG pathway map 00941 is adapted here from http://www.kegg.jp/kegg/kegg1.html. The KEGG database has been described previously^60–62^.

One of the study reported that butin has aromatase inhibitory action whereas butein shows its effect against breast and lung cancer^36–38^. Pinostrobin has been reported to have antiviral property^39^ and it also possesses antioxidant and chemoprotective activity^40^. Naringenin, a flavonone flavonoid, has been shown to possess marked antioxidant and anti-inflammatory properties^41^. Naringenin has been reported to have a protective effect on carbon tetracholride induced acute nephrotoxicity in mouse models^42^. Naringenin is reported to be an effective chemotherapeutic agent for prostate cancer^43^. It has also been reported that *S. trilobatum* possesses neuroprotective role against pathology of Parkinson’s disease^44^. Galangin is considered to be the potential candidate for new drugs against Alzheimer’s disease^45^. It also possesses hepatoprotective and anticancer properties^46,47^. Quercetin is one of the important flavonoids reported in our dataset, it possess antioxidant, also shows anti-carcinogenic and hepatoprotective properties^48^. Luteoforol, quercetin, and myricetin are reported to have anti microbial properties^49,50^. The flavonol dihydrofisetin (fustin) is reported to show protective role in neuronal cell death^51^. The Kaempferol also identified in our dataset is a well-known phytoestrogen reported to induce osteoblastic differentiation^52^. Kaempferol is also reported to possess hepatoprotective, antioxidant and anticancer effects^53–55^. Dihydroquercetin (Taxifolin) reported in our data acts as a potential chemopreventive agent^56^. Cyanidin is also reported as potent inhibitor of EGFR, shutting off downstream signalling cascades^57^ (Fig. 6). A summary of major genes involved in phenylpropanoid and flavonoid pathways has been presented in Table 3.

**Table 3:** Major genes involved in phenylpropanoid and flavonoid biosynthesis pathways identified from *Solanum trilobatum* leaf transcriptome.

Gene name	EC Number	Unigene ID	Unigene length	FPKM value	TPM value	No. Of unigenes
**Phenylpropanoid biosynthesis pathway genes**						
Phenylalanine Ammonia Lyase (PAL)	4.3.1.24 4.3.1.25	TRINITY_DN24381_c0_g1_i1 TRINITY_DN26588_c0_g1_i1 TRINITY_DN26588_c0_g1_i2 TRINITY_DN9424_c0_g1_i1	2568 2489 2413 1982	8.85 35.51 35.51 3.39	13.97 56.01 56.01 5.34	4
Cinnamate 4-hydroxylase/trans-cinnamate 4-monooxygenase	1.14.13.11	TRINITY_DN26248_c0_g2_i2 TRINITY_DN26248_c0_g2_i3	1921 1998	16.64 16.64	26.25 26.25	2
Cinnamoyl-CoA reductase	1.2.1.44	TRINITY_DN23155_c0_g1_i1 TRINITY_DN23155_c0_g1_i2	1349 1430	4.44 4.44	7 7	2
**Flavonoid biosynthesis pathway genes**						
Flavonoid 3′, 5′-hydroxylase	1.14.13.88	TRINITY_DN20157_c0_g1_i1 TRINITY_DN20157_c0_g1_i2	1756 1329	1 1	1.58 1.58	2
Flavonoid 3′ -monooxygenase	1.14.13.21	TRINITY_DN62729_c0_g1_i1	1796	1.12	1.76	1
Chalcone synthase	2.3.1.74	TRINITY_DN18047_c0_g1_i1 TRINITY_DN18047_c0_g2_i1 TRINITY_DN45084_c0_g1_i1	1334 1354 925	1.25 0.84 1.29	1.97 1.32 2.04	3
Chalcone isomerase	5.5.1.6	TRINITY_DN62987_c0_g1_i1	1272	6.98	11.02	1
Flavonol synthase	1.14.11.23	TRINITY_DN9434_c0_g1_i1	1307	6.88	10.85	1
Leucoanthocyanidin dioxygenase	1.14.11.19	TRINITY_DN25629_c0_g1_i2 TRINITY_DN25629_c0_g1_i4	1695 1454	1.47 1.47	2.31 2.31	2
Naringenin 3-dioxygenase	1.14.11.9	TRINITY_DN18592_c0_g3_i1	1314	2.63	4.16	1
Coumaroylquinate 3′-monooxygenase	1.14.13.36	TRINITY_DN20637_c0_g1_i1 TRINITY_DN9604_c0_g1_i1 TRINITY_DN9604_c0_g2_i1	1757 1113 826	6.73 1.33 1.12	10.62 2.1 1.77	3
Flavonone 4-reductase	1.1.1.234	TRINITY_DN65302_c0_g1_i1 TRINITY_DN41921_c0_g1_i1	625 683	0.78 0.56	1.24 0.89	2

### Discovery of simple sequence repeats (SSRs)

**Figure 7:**
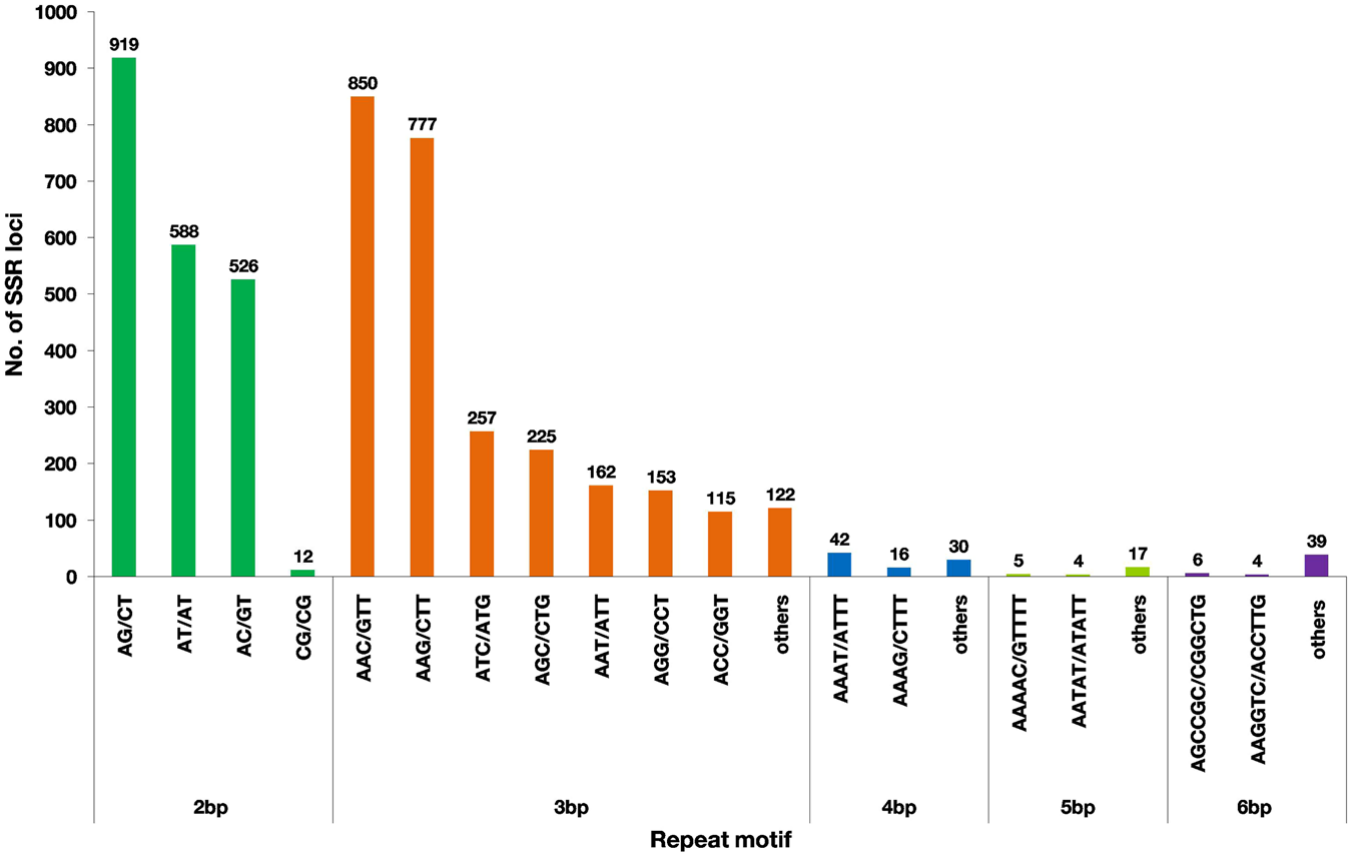
Summary of SSR types identified in *Solanum trilobatum* leaf transcriptome.

Microsatellites or SSRs are tandem repeats of DNA sequences abundantly present throughout the genome^58^ and are widely useful for molecular- assisted selection (MAS) in plant breeding and improvement programs. We analyzed 128,934 assembled transcripts from *S. trilobatum* using MISA tool and a total of 13,262 SSRs were identified including 561 SSRs in compound formation. Among the analyzed sequences 11,727 contained SSRs and 1,303 sequences showed the presence of more than one SSR. The statistical distribution of SSRs is given in Table 4. The most abundant repeat motif was found to be tri-nucleotide repeats with 2661(54.65%) followed by di-nucleotide repeats 2045 (42%), tetra-nucleotide 88 (1.81%), penta-nucleotide 26 (0.53%) and hexa-nucleotide 49 (1.01%). SSRs with five tandem repeats (1,840) were most common in *S. trilobatum* and are followed by six repeats (1,376) and seven repeats (665). Among di-nucleotide repeats, AG/CT (44.94%) followed by AT/AT (28.75%), AC/GT (25.72%), CG/CG (0.59%). In case of tri-nucleotide repeats highest frequency occurred in AAC/GTT (31.94%), followed by AAG/CTT (29.20%), ATC/ATG (9.66%), AGC/CTG (8.45%) and other motifs were almost uniformly distributed (Fig. 7).

**Table 4:** Distribution and frequency of EST-SSRs identified in *Solanum trilobatum*.

Motif length	Repeat Numbers							Total	%
	5	6	7	8	9	10	>10		
Di	0	818	431	273	163	106	254	2045	42.00%
Tri	1725	533	224	103	29	11	36	2661	54.65%
Tetra	76	9	2	1	0	0	0	88	1.81%
Penta	25	1	0	0	0	0	0	26	0.53%
Hexa	14	15	8	8	1	3	0	49	1.01%
Total	1840	1376	665	385	193	120	290		
%	37.79%	28.26%	13.66%	7.91%	3.96%	2.46%	5.96%

### Transcription factors from *S. trilobatum*

**Figure 8:**
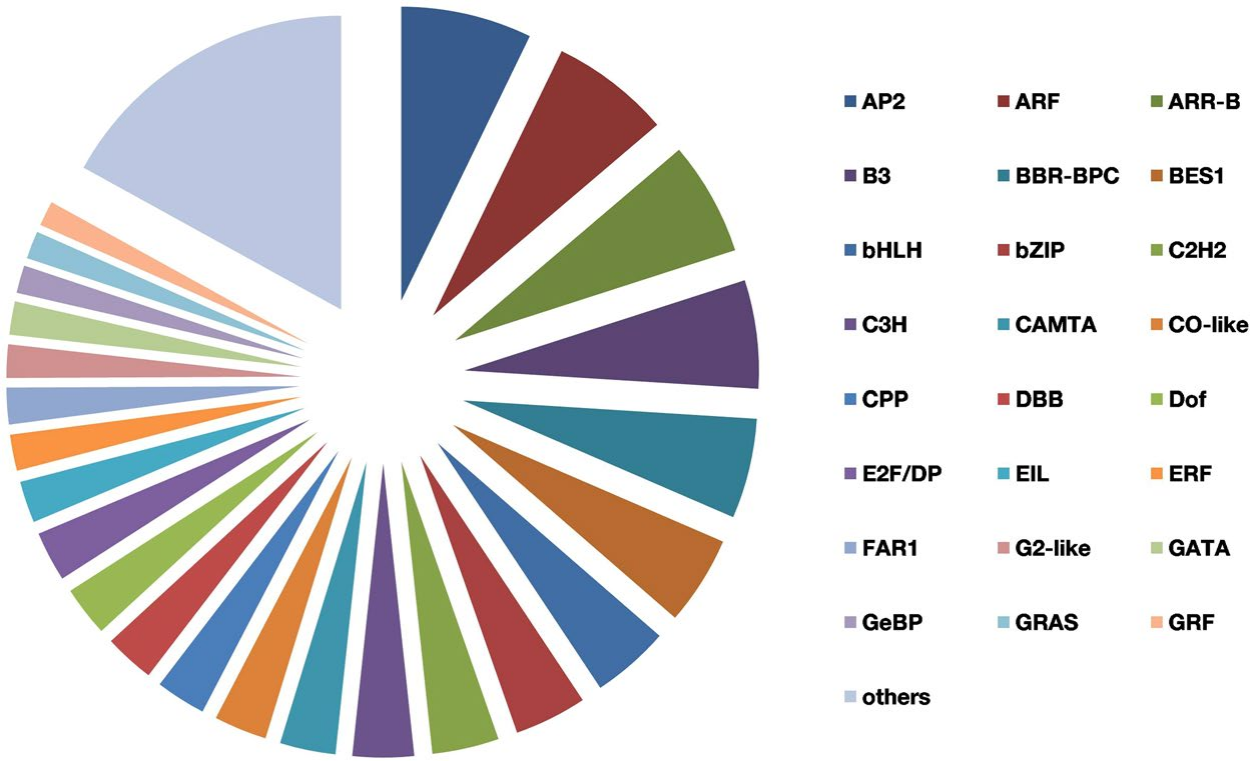
Transcription factors identified from *Solanum trilobatum* leaf transcriptome.

Transcription factors (TFs) play a crucial role in regulation of secondary metabolites by regulating the expression of related genes of biosynthetic pathways. The identification of such TFs will benefit us in understanding gene regulatory networks. TFs known to regulate plant secondary metabolism include R2R3-MYB, bHLH proteins like CrMYC2, AP2/ERF family proteins, WRKY, NAC, DOF, bZIP, HD-ZIP and TFIIIA zinc finger TFs^59^. We identified a total of 654 unitranscripts representing 48 TF families. Among the represented families, AP2 family TFs (47 unitranscripts) were the most abundant followed by ARF (43), ARR-B (41), B3 (39), BBR-BPC (36), BES1 (32), bHLH (28), bZIP (26) and C2H2 (24). Among the annotated TF unigenes, TFs related to Secondary metabolism were AP2, bHLH, bZIP, Dof, MYB, MYB related, WRKY and ZF-HD (Fig. 8) (See Supplementary Table S5).

### Transcript quantification

The estimation of transcript abundance or expression levels of *de novo* assembled unigenes from *S. trilobatum* leaf transcriptome was calculated based on FPKM and TPM values using RSEM. The FPKM and TPM values for each unigene and isoforms of our data set are given in Table S6 (See Supplementary Information). The flavonoid biosynthesis pathway is highly represented as per our data analysis and also includes phenylpropanoid biosynthesis pathway as its backbone. So, understanding the transcript abundance of unigenes involved in phenylpropanoid and flavonoid biosynthesis pathways would be of great importance. In phenylpropanoid pathway, the TPM values of key enzymes involved are 56.01, 26.25 and 7 for Phenylalanine Ammonia Lyase (PAL), Cinnamate 4-hydroxylase/trans-cinnamate 4-monooxygenase and Cinnamoyl-CoA reductase respectively. In case of flavonoid biosynthesis pathway, the key genes with highest TPM values are Chalcone isomerase, Flavonol synthase and Coumaroylquinate 3′-monooxygenasewith 11.02, 10.85, 10.62 respectively and the gene encoding for flavonoid 3′, 5′-hydroxylaseobtained lowest TPM value of 1.58 (Table 3). The list of top most abundant unigenes for *de novo* assembled *S. trilobatum* transcriptome is given in Table 5. The list includes genes mostly from chloroplast, and our data set is a leaf transcriptome so it’s expected to have these genes abundantly reflected in our transcriptome data analysis.

**Table 5:** Top 10 most abundant genes from leaf tissue of *Solanum trilobatum*.

Transcript ID	Gene Name	TPM	FPKM
TRINITY_DN26613_c2_g2	Photosystem II D1 (chloroplast)	424070.40	268815.80
TRINITY_DN26771_c1_g1	Ribulose Bisphosphate Carboxylase Small Chloroplastic	18400.13	11663.74
TRINITY_DN26916_c0_g3	ATP Synthase CF1 beta subunit (chloroplast)	16994.00	10772.40
TRINITY_DN25001_c0_g1	Hypothetical Protein POPTR_1605s00200g	13786.36	8739.10
TRINITY_DN26771_c4_g4	Ribulose Bisphosphate Carboxylase small chain Chloroplastic	13279.73	8417.94
TRINITY_DN26771_c4_g3	Ribulose Bisphosphate Carboxylase small chain Chloroplastic	12732.03	8070.76
TRINITY_DN25898_c0_g1	Predicted Protein, partial	8805.54	5581.78
TRINITY_DN26549_c1_g1	Photosystem II CP47 reaction center -like	6956.46	4409.66
TRINITY_DN27071_c3_g8	Photosystem I P700 Apo A2 (chloroplast)	6124.31	3882.16
TRINITY_DN27083_c0_g1	Ycf68 (chloroplast)	5972.38	3785.85

### Validation by reverse transcription PCR

**Figure 9:**
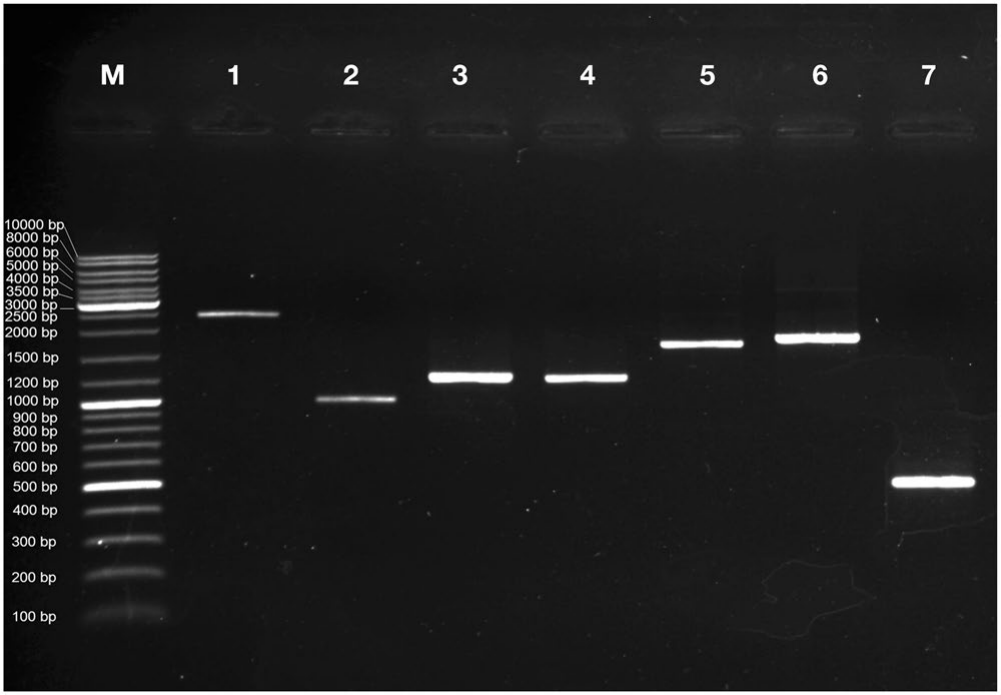
Reverse Transcription PCR analysis of selected unigenes of *Solanum trilobatum* leaf transcriptome. M is GeneRuler DNA ladder mix (Thermo Fisher Scientific, Waltham, MA), Lane 1–7 are amplicons of Phenylalanine ammonia lyase (2396 bp), Chalcone isomerase (998 bp), Flavonol synthase (1227 bp), Naringenin 3-dioxygenase (1215 bp), Coumaroylquinate 3′-monooxygenase (1673 bp), Squalene monooxygenase (1785 bp) and Actin (480 bp) respectively.

The secondary metabolite genes of *S. trilobatum* were selected for performing Reverse Transcription-PCR to validate the assembled unigenes from leaf transcriptome. We selected a total of seven genes involved in secondary metabolite biosynthesis such as Phenylalanine ammonia lyase (EC: 4.3.1.24) from Phenylpropanoid biosynthesis pathway; Chalcone Isomerase (EC: 5.5.1.6), Flavonol synthase (EC: 1.14.11.23), Naringenin-3-dioxygenase (EC: 1.14.11.9) and Coumaroylquinate 3′-monooxygenase (EC: 1.14.13.36) from flavonoid biosynthesis pathway; Squalene monooxygenase (EC: 1.14.14.17) from Steroid biosynthesis pathway. We performed Reverse transcription PCR for above mentioned unigenes with Actin as positive control (Fig. 9). Experimental confirmation of the gene expression data authenticates the functionally annotated transcriptome assembly.

### Gene expression analysis of *Solanum trilobatum* leaf tissue

**Figure 10:**
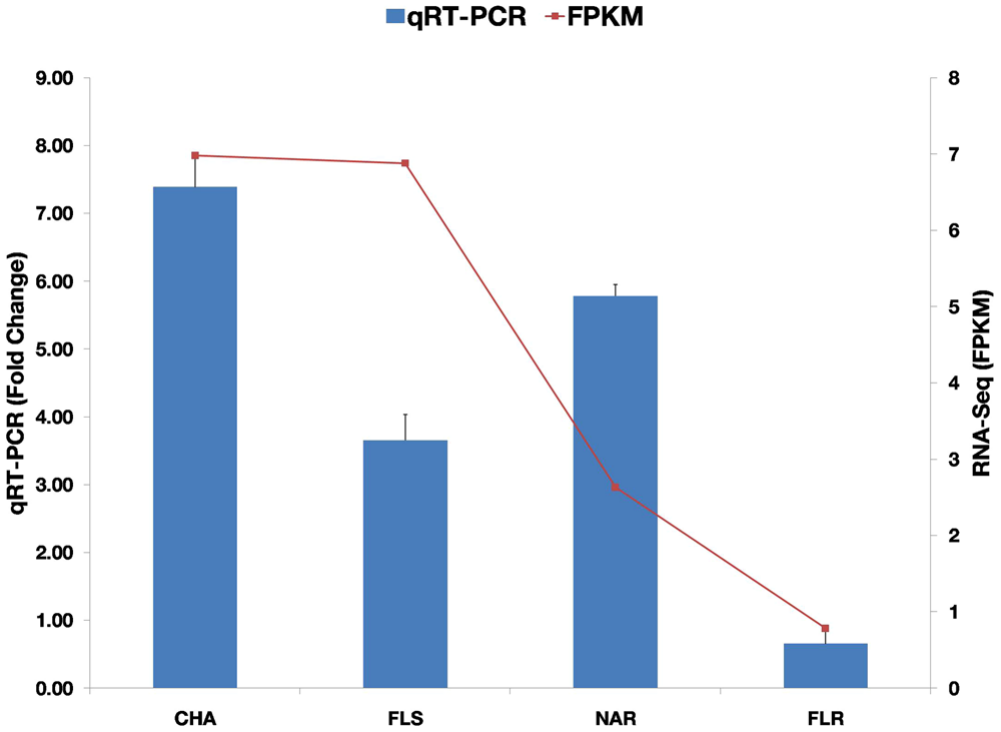
Validation of gene expression by qRT-PCR. CHA is Chalcone Isomerase, FLS (Flavonol Synthase), NAR (Naringenin 3-dioxygenase) and FLR is Flavonone 4- reductase.

The qRT-PCR was introduced to analyze the expression pattern of selected flavonoid biosynthesis genes and to validate the transcriptome assembly in *S. trilobatum* leaf. The Selected transcripts included Chalcone Isomerase (EC: 5.5.1.6), Flavonol Synthase (EC: 1.14.11.23), Naringenin 3-dioxygenase (EC: 1.14.11.9) and Flavonone 4-reductase (EC: 1.1.1.234). Based on our qRT-PCR results, the selected genes displayed different expression profiles, where Chalcone isomerase, Flavonol synthase and Naringenin 3-dioxygenase showed significant up-regulation in leaf tissue when compared to stem as control, with Chalcone isomerase showing maximum up-regulation and also Flavonone-4 reductase showing significant down regulation in the leaf tissue (See Supplementary Table S7). The qRT-PCR expression profiles of selected genes showed significant agreement with our transcriptomic data hence validating the *de novo* assembly of *Solanum trilobatum* leaf tissue (Fig. 10).

## Conclusion

The main aim of our study was to analyze the transcriptome of *Solanum trilobatum* using Illumina high throughput sequencing platform. In order to facilitate molecular research in *Solanum trilobatum*, we characterized the leaf transcriptome for identification of unitranscripts involved in biosynthesis of secondary metabolites, since several medicinal attributes are affiliated to leaf tissue of this plant. Our results suggested that Flavonoid biosynthesis pathway is highly represented in the leaf transcriptome and is possibly contributing for the medicinal attributes of this plant. The predicted biosynthetic pathway is going to serve as lead for identification and isolation of medicinally important phytocompounds. We have also quantified the expression levels of transcripts for various important metabolic pathways. We have validated the *de novo* assembly by amplifying randomly selected unigenes by Reverse Transcription PCR method and also performed gene expression analysis of selected key genes from flavonoid biosynthesis pathway by qRT-PCR. This is the first report on leaf transcriptome assembly and analysis of *Solanum trilobatum* and it will serve as an important resource for studying molecular mechanisms involved in biosynthesis of its medicinal compounds.

## Electronic supplementary material

Table S1

Table S2

Table S3

Table S4

Table S5

Table S6

Table S7
